# Polymorphism of the CYP2C9 and VKORC1 genes: a comment

**DOI:** 10.1590/1677-5449.210108

**Published:** 2021-09-16

**Authors:** Rujittika Mungmunpuntipantip, Viroj Wiwanitkit

**Affiliations:** 1 Private Consultant, Bangkok, Thailand.; 2 Padmashree Dr. DY Patil University, Pune, India.

Dear editor,

We would like to share ideas on “Polymorphism of the CYP2C9 and VKORC1 genes in patients on the public health system of a municipality in Southern Brazil.^1^” Tozato et al. studied polymorphisms of the CYP2C9 and VKORC1 genes, warfarin, and Time in Therapeutic Range (TTR) and concluded that “An association was observed between the polymorphisms and the warfarin doses taken by the patients. However, there was no association with adverse events or the time spent within the therapeutic range in this sample.^1^” The study drew conclusions that were not supported by its findings. It should only have concluded on the genetic epidemiology of polymorphisms. There are many confounding factors. An important consideration is the effect of other polymorphisms that are not assessed in the present study. Examples of these polymorphisms are CYP4F2 V433M, ABCB1, PROC, EPHX1, and SETD1A.^2^^,^ 3
