# Supplementary material for: Polymorphism of the CYP2C9 and VKORC1 genes: a comment
Source: J Vasc Bras. 2021 Sep 16;20:e20210108. doi: 10.1590/1677-5449.210108 (PMC8483011; doi:10.1590/1677-5449.210108)
Supplement: Related texts

# Reply

**Authors:** Christiane de Fátima Colet

Dear Editor,

We agree with the point made in this Letter to the Editor. As we mentioned in our article, some associations that have been identified in earlier publications were not detected in our study and we believe that this may be because the population analyzed in our study was insufficient for certain causal inferences. We also agree that the clinical response is the result of the activity of multiple genes, as stated in the Letter, but it was not possible to include all of these genes for financial reasons, so we chose those that have the greatest influence on warfarin efficacy according to the literature.
